# SKIP Silencing Decreased Disease Resistance Against *Botrytis cinerea* and *Pseudomonas syringae* pv. *tomato* DC3000 in Tomato

**DOI:** 10.3389/fpls.2020.593267

**Published:** 2020-12-14

**Authors:** Huijuan Zhang, Longfei Yin, Fengming Song, Ming Jiang

**Affiliations:** ^1^Life Science Collegue, Taizhou University, Taizhou, China; ^2^National Key Laboratory for Rice Biology, Institute of Biotechnology, Zhejiang University, Hangzhou, China

**Keywords:** *SKIP*, *B. cinerea*, *Pst* DC3000, resistance, ROS

## Abstract

SKIP, a component of the spliceosome, is involved in numerous signaling pathways. However, there is no direct genetic evidence supporting the function of SKIP in defense responses. In this paper, two *SKIPs*, namely, *SlSKIP1a* and *SlSKIP1b*, were analyzed in tomato. qRT-PCR analysis showed that the *SlSKIP1b* expression was triggered via *Pseudomonas syringae* pv. *tomato* (*Pst*) DC3000 and *Botrytis cinerea* (*B. cinerea*), together with the defense-associated signals. In addition, the functions of *SlSKIP1a* and *SlSKIP1b* in disease resistance were analyzed in tomato through the virus-induced gene silencing (VIGS) technique. VIGS-mediated *SlSKIP1b* silencing led to increased accumulation of reactive oxygen species (ROS), along with the decreased expression of defense-related genes (DRGs) after pathogen infection, suggesting that it reduced *B. cinerea* and *Pst* DC3000 resistance. There was no significant difference in *B. cinerea* and *Pst* DC3000 resistance in TRV-*SlSKIP1a*-infiltrated plants compared with the TRV-*GUS*-silencing counterparts. As suggested by the above findings, *SlSKIP1b* plays a vital role in disease resistance against pathogens possibly by regulating the accumulation of ROS as well as the expression of DRGs.

## Introduction

The splicing process is completed by spliceosome, which can be classified as two types, including major spliceosome and minor spliceosome (Moore and Proudfoot, 2009; Turunen et al., 2013). SKIP, one of the splicing factors and important components of spliceosome, has possessed several conserved domains (including the SNW/SKI-interacting protein, SKIP) and the specific motifs (Folk et al., 2004; Bres et al., 2009; Chen et al., 2011; Wang et al., 2012). The C terminal plays a vital role in protein stability, while the SNW domain is necessary for the biochemical activity. In Arabidopsis, the SNW domain can integrate into spliceosome in the meantime of interacting with the Paf1 complex (Li et al., 2016). The interaction of SMP1/2 with SKIP facilitates the recruitment of second-step splicing factors into the Arabidopsis spliceosome (Liu et al., 2016).

SKIP is involved in splicing in an either direct or indirect way. In mammals and yeast, SKIP is a component of the 35S-U5snRNP complex, which participates in the common RNA splicing directly (Albers et al., 2003). Growing evidence shows that SKIP is involved in transcription regulation and RNA splicing through interacting with different proteins and thus takes parts in regulating several signaling pathways. For example, SKIP protein participates in at least five signaling pathways in human, including the steroid hormone (Zhang et al., 2001, 2003), TGF-β (Barry et al., 2003), MyoD (Kim et al., 2001), Notch (Zhou et al., 2000; Laduron et al., 2004), and E2F/pRb (Prathapam et al., 2002) pathways.

In addition to RNA splicing, SKIP exerts its functions in numerous steps, such as transcription elongation and transport of mature mRNA. Many species harbor the homologous SKIP protein in nucleus that is between 60 and 80 kDa. However, SKIP in different species has different functions. For instance, in yeast, the weak mutation of the SKIP homolog Prp45 has defects in the splicing of *ACTIN* and other genes, which leads to the fatal potent mutation of Prp45 and the growth with temperature sensitivity (Figueroa and Hayman, 2004; Bessonov et al., 2008; Gahura et al., 2009). In yeast, SKIP, one of the transcription factors (TFs), helps to modulate gene expression patterns (Lim et al., 2010). In drosophila, *Bx42* involved in transcription regulates ecdyson, the Notch signaling pathway, nervous system development, and organ formation (Wieland et al., 1992; Negeri et al., 2002; Ivanov et al., 2004). In nematode, *CeSKIP* exerts an essential role in individual survival and embryonic development (Kostrouchova et al., 2002; Piano et al., 2002; Kamath et al., 2003; Simmer et al., 2003; Rual et al., 2004; Sonnichsen et al., 2005). Recently, the SKIP functions have been gradually explored, but mainly in Arabidopsis. SKIP shows physical interaction with SR45 (an SR protein specific to plant) for regulating the biological clock. Mutations of the *skip*-1 gene will lead to a phenotype of a prolonged clock period through changing alternative splicing (AS) in PSEUDO-RESPONSE REGULATOR 7 (*PRR7*) together with *PRR9*, both of which are related to the oscillator morning loop. As indicated by this result, *SKIP* participates in regulating the genes associated with the biological clock of Arabidopsis at the post-transcription level (Wang et al., 2012). Apart from the defects of the biological clock, the *skip-1* plant also exhibits the pleiotropic phenotype, such as the early blossoming. Additionally, it is still unclear about how *SKIP* suppresses floral transition at a molecular or biochemical level, although there are several hypotheses. Cao et al. (2015) reported that, in Arabidopsis, *SKIP* activated the transcription of *FLC* to modulate its blossoming through interacting with the *Paf1* complex. In Arabidopsis, SKIP regulates the blossoming time by AS of SEF pre-mRNA (Cui et al., 2017), while *AtSKIP* plays a role of an adjuster between the light signal transduction pathway and cytokinin and thus regulates the cytokinin-associated leaf growth (Zhang X. et al., 2014).

Nowadays, it has been reported that SKIP plays a certain role in the response to stress in a variety of plant, such as Arabidopsis, rice, and maize. The expression level of *SKIP* can be triggered via salt, abscisic acid (ABA), and mannitol. In the germination process of Arabidopsis, *SKIP* overexpression leads to abiotic stress tolerance, while *SKIP* downregulation reduces the abiotic stress tolerance. *AtSKIP* participates in the ABA signal transduction pathway, which renders resistance to salt or osmotic stress by controlling gene AS of Arabidopsis (Lim et al., 2010; Feng et al., 2015). *OsSKIPa*, which results in fatal defect in the *SKIP* homolog knockout mutant of yeast, shows positive modulation on cell growth, viability, and resistance to stress in rice through regulating diverse genes associated with stress at the transcription level (Hou et al., 2009). Besides, interaction of *OsSKIP* and *OsCYP18-2* also exerts a vital part in regulating genes associated with stress at the post-transcription or transcription level and enhancing drought resistance (Lee et al., 2015). In addition, the *ZmSKIP* overexpression plants with increased ABA content show significantly increased resistance to drought compared with the controls, suggesting that *ZmSKIP* is involved in the regulation of drought resistance by modulating drought-associated gene expression (Niu, 2012; Wei et al., 2015). *GhSKIP35* has a certain function in the resistance to verticillium wilt in *Gossypium hirsutum* (Liu, 2015). *GmGBP1*, the human ski interacting protein homolog of soybean, can modulate blossoming together with stress resistance of Arabidopsis by regulating the scavenging activity for reactive oxygen species (ROS) (Zhang et al., 2013).

This research aimed to analyze the functions of *SKIP* genes in tomato to resist against *Botrytis cinerea* as well as *Pseudomonas syringae* pv. *tomato* (*Pst*) DC3000, the necrotrophic fungal and the (hemi)-biotrophic bacterial pathogens, separately, using virus-induced gene silencing (VIGS). It was shown in this study that the VIGS-mediated silencing of *SlSKIP1b* resulted in the accumulation of more ROS, but decreased the levels of defense-related genes (DRGs) in the case of pathogen infection, thereby attenuating *B. cinerea* and *Pst* DC3000 resistance. These findings demonstrated that the *SKIP* genes play vital parts in regulating the anti-pathogen response of tomato.

## Materials and Methods

### Plant Cultures as Well as Treatments

Two tomato varieties (*Solanum lycopersicum*), Suhong2003 as well as MicroTom, were employed in this research. Suhong2003 was used in all experiments except for the whole plant disease assays against *B. cinerea* that used the cultivar MicroTom. Tomato seedlings were grown in a material mixture within a greenhouse. Besides, seedlings of 2 and weeks old were utilized to carry out VIGS assays and to analyze gene expression after pathogen inoculation and treatments with defense-related signal molecules, respectively. Typically, the treatments with defense-related signal molecules were done by spraying MeJA, ACC, and SA (all at 100 μM and from Sigma-Aldrich), and water was used as control. At the designated time points after treatment, the leaves were collected.

### Pathogen Infection Together With Disease Assays

In this study, *B. cinerea* infection in tomato plants was completed by two approaches (Li et al., 2014; Zhang et al., 2020). In brief, after collecting spores, their densities were tuned to 1 × 10^5^ spores/mL. For the detached leaf disease assays, leaf samples with full expansion were collected from the 6-week-old VIGS agroinfiltrated plants, and put on the cheesecloth pre-immerged within the distill sterilized water in trays. Each side of the leaves was inoculated by a drop of 2.5 μL spore suspension, followed by disease development in high humidity. At 4 days later, the lesion size in those infected leaf samples was recorded. In the whole plant disease assays, spore suspension was sprayed onto tomato plants until it evenly covered the leaf surface. Afterward, those infected plants were then put into a high-humidity environment. At 4 days after inoculation, photographs were taken for the phenotype. Then, after collecting leaves at the designated time points, the fungus quantity and gene levels were analyzed. qRT-PCR was adopted to define fungal growth by *B. cinerea BcActinA* gene expression.

Plants were inoculated with *Pst* DC3000 according to the following steps (Li et al., 2014): after harvesting and resuspending bacteria into MgCl_2_ (10 mM) to OD_600_ = 0.0002, all leaves were immersed into the bacterial suspension using the 0.04% Silwet L-77, followed by 1.5 min of negative pressure treatment at −40 kPa. The phenotype was photographed at 4 days following infection. Besides, leaves were harvested to analyze specific gene levels as well as bacterial growth. To measure the bacterial growth, 70% ethanol was used to sterilize leaf discs for 10 s, then sterile water was utilized to wash them for thrice, followed by grinding within the 10 mM MgCl_2_ solution (200 μL) until a homogenate was obtained. Later, the homogenate was diluted with 10 mM MgCl_2_ at a ratio of 1:10 to different concentrations, cultured in the King’s B solid medium for 3 days, and the colonies were recorded.

### Characterization of SlSKIP Genes

Using the BlastP program, the tomato genome database was searched at http://solgenomics.net using those featured Arabidopsis *AtSKIP* as queries. Afterward, those obtained sequences of *SlSKIPs* nucleotides and amino acids (AAs) were downloaded.

### RNA Extraction Along With qRT-PCR

The Trizol reagent (Invitrogen, Shanghai, China) was used to extract total RNA according to specific protocol. The PrimeScript RT reagent kit (TaKaRa, Dalian, China) was used for reverse transcription following specific instructions to synthesize cDNAs, which served as the templates for PCR and qRT-PCR. In this study, the CFX96 real-time PCR assay system (Bio-Rad, Hercules, CA, United States) was used for qRT-PCR. Dissociation curves were used to verify that the amplified production was single in PCR. Target gene transcript expression was shown as relative transcript expression to an *Actin* gene in tomato. The 2^–△^^△^^CT^ approach was applied in calculating the relative gene expression level according to previous description. Table 1 lists those gene-specific primers adopted for qRT-PCR.

**TABLE 1:** The list of primer sequence of the genes in this article.

Primers	Sequences (5′–3′)	Size (bp)
*SKIP1a-vigs-F*	GTC TCTAGA TGGCATCTCTCAAGGAGCT	351
*SKIP1a-vigs-R*	GTC TCTAGA GTTACTGTGGACGAACACGGT	
*SKIP1b-vigs-F*	GTC TCTAGA GTTACTGTGGACGAACACGGT	349
*SKIP1b-vigs* -R	AGT CTCGAG GTACCTTCTTGTGCTTGAACT	
*SKIP1a-RT-F*	TAGTGGAGGCACCATGAAGG	113
*SKIP1a-RT-R*	GCTGGCAGTGGAAGACAATT	
*SKIP1b-RT-F*	TACTTACGGAGAGCAGCAACA	112
*SKIP1b-RT-R*	AGCCTCAAATTCCACAGGTCTA	
*BcActin*-qRT-F	CGTCACTACCTTCAACTCCATC	107
*BcActin*-qRT-R	CGGAGATACCTGGGTACATAGT	
*SlActin*- qRT-F	CCAGGTATTGCTGATAGAATGAG	113
*SlActin*- qRT-R	GAGCCTCCAATCCAGACAC	
*SlPR1b*-qRT-F	TTTCCCTTTTGATGTTGCT	96
*SlPR1b*-qRT-R	TGGAAACAAGAAGATGCAGT	
*SlPRP2*-qRT-F	CGATCTAAATTGATTTCATAGTACG	116
*SlPRP2*-qRT-R	TCGTGAAGGATATACAAAATACA	
*SlLapA*-qRT-F	GGGACTAATGATGTTTGGAA	109
*SlLapA*-qRT-R	GTGGCAATTTTATTTAGGCA	
*SlPIN2*-qRT -F	CATCTTCTGGATTGCCCA	106
*SlPIN2*-qRT -R	ACACACAACTTGATGCCCAC	

### Construction of the VIGS Vector and Agroinfiltration

Fragments of 300–400 bp in sizes for *SlSKIPs* were amplified by PCR with respective pairs of gene-specific primers (Table 1). The amplified PCR products were digested with corresponding restriction enzymes (*Xba*I/*Xho*I) and cloned into TRV2, yielding recombinant plasmids TRV-SlSKIP1a and TRV-SlSKIP1b. After confirmation by sequencing, the correct recombinant plasmids were transformed into *Agrobacterium tumefaciens* strain GV3101 by electroporation and positive clones were selected by colony PCR for VIGS assays. Agrobacteria carrying TRV-SlSKIP1a or TRV- SlSKIP1b were grown in YEP liquid medium with 50 μg/mL kanamycin, 50 μg/mL rifampicin, and 25 μg/mL gentamicin in a shaker until OD_600_ reached to 0.8∼1.0. Agrobacterial cells were collected by centrifugation and resuspended in infiltration buffer containing 10 mM MgCl_2_, 10 mM MES (pH5.7) and 200 μM acetosyringone, and the bacterial concentrations in suspensions were adjusted to OD_600_ = 1.5. The agrobacteria carrying TRV-SlSKIP1a or TRV-SlSKIP1b were mixed with the same volume of agrobacteria carrying TRV1, and the mixtures were incubated for 3 h at room temperature. The mixed agrobacterial suspension was separately infiltrated into the abaxial surface of the 2-week-old seedlings using a 1-mL needleless syringe (Liu et al., 2002). A group of tomato seedlings were infiltrated with agrobacteria harboring a construct of TRV-PDS (*Phytoene desaturase*) and used as positive controls for silencing evaluation of the VIGS procedure (data showed in Supplementary Material). The agroinfiltrated plants were allowed to grow for 4 weeks in a growth room under the same conditions as mentioned above and then used for different experiments.

### Detection and Measurement of H_2_O_2_

The DAB staining method was utilized to detect H_2_O_2_ accumulation within the leaf tissues. After *B. cinerea* (0 and 24 h) and *Pst* DC3000 (0 and 48 h) inoculation, leaves were harvested, respectively. After 3 h of immersion into the 1-mg/ml DAB solution (pH 3.8), the leaf samples were boiled in 95% ethanol until the chlorophyll was completely removed. Finally, a digital camera was utilized to visualize H_2_O_2_ accumulation in those stained leaf samples. The H_2_O_2_ measurement was done by an H_2_O_2_ Kit (Jiancheng, Nanjing, China). The content of H_2_O_2_ was calculated by the formula: (OD value of the measured sample − OD value of the blank)/(OD value of a standard solution − OD value of the blank × 163 mmol/L)/concentration of the protein of tissue.

### Experiment Design and Data Analysis

All experiments were repeated independently three times. More than 10 plants were used in each of independent experiments such as disease assay with *B. cinerea* or *Pst* DC3000. Data obtained from three independent experiments were subjected to statistical analysis according to the Student’s *t*-test. The probability values of *p* < 0.05 were considered as significant difference between treatments and their corresponding controls.

## Results

### Characterization of SlSKIP Genes in Tomato

Using the characterized Arabidopsis *AtSKIP* genes as queries, a tomato genomic database was searched by Blastp analysis, and two loci were identified in tomato genome, which were named as *SlSKIP1a* (XM_004251580.4) and *SlSKIP1b* (XM_004250540.4) (the information about *SlSKIPs* can be seen in Supplementary Data). Moreover, ESTs together with the potential full-length cDNAs of *SlSKIP*s were discovered against tomato genomic database as well as NCBI GenBank database, separately, which indicated the constitutive expression of *SlSKIP*s in tomato. The ORFs of *SlSKIPs* were cloned and sequenced, which found the totally same *SlSKIPs* ORF sequences with those predicted ORF sequences.

### Expression Models of SlSKIPs With Pathogen Inoculation and Treatments With Defense-Related Signal Molecules

**FIGURE 1:**
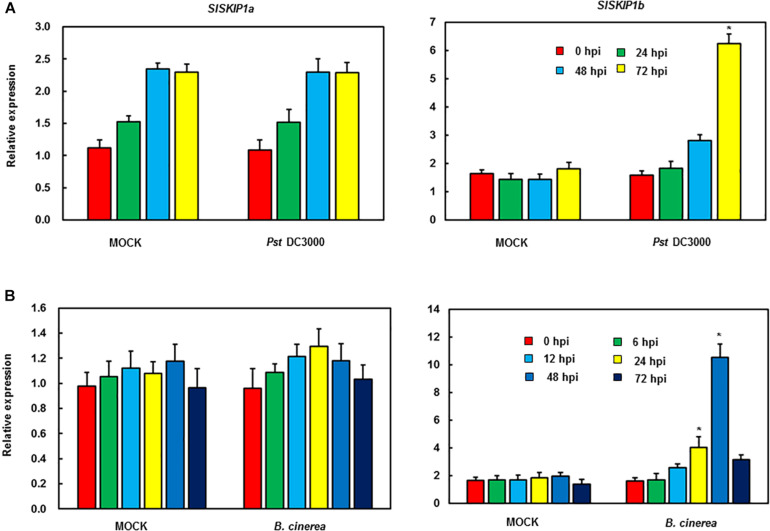
*SlSKIP* expression responding to *B. cinerea* together with *Pst* DC3000 infection. **(A)** *SlSKIP* gene expression patterns responding to *Pst* DC3000 infection. **(B)** *SlSKIP* gene expression patterns responding to *B. cinerea* infection. Data presented are the means ± SD from three independent experiments with biological distinct samples and * above the columns indicate significant differences at *p* < 0.05 level.

**FIGURE 2:**
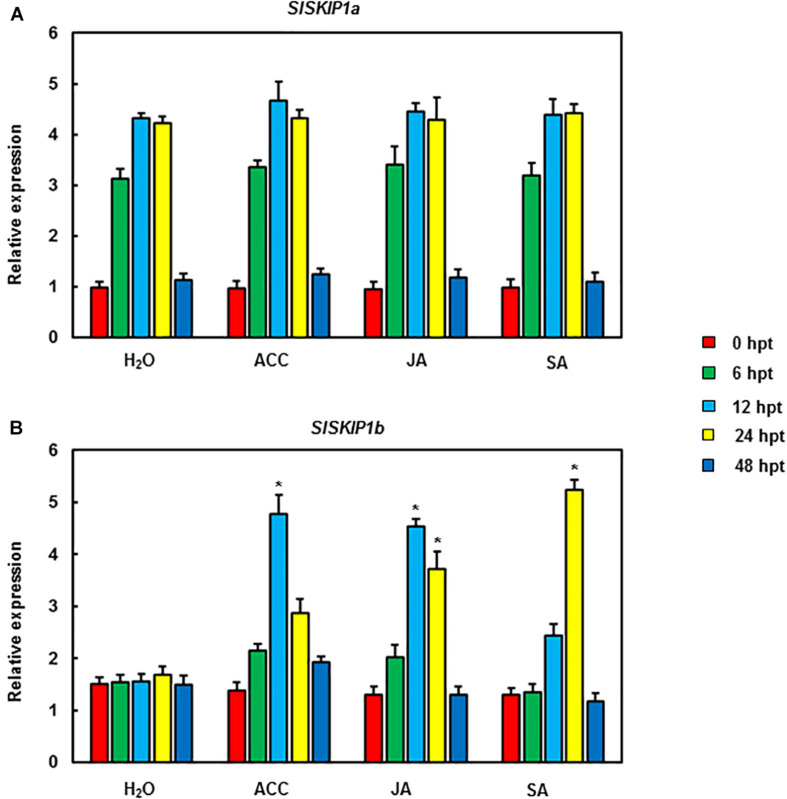
*SlSKIP* expression levels responding to treatments with defense-related signal molecules. *SlSKIP* gene expression models responding to defense-related signal molecules treatments were analyzed. To be specific, 100 μM of SA, MeJA, and ACC was used to treat tomato plants by foliar spraying; alternatively, a similar amount of solution with no abovementioned signal molecules was used as control. Later, the leaves were harvested at the indicated time points to analyze gene expression through qRT-PCR. Data presented are the means ± SD from three independent experiments with biological distinct samples and * above the columns indicate significant differences at *p* < 0.05 level. **(A)** The expression level of SlSKIP1a with the treatments of defense-related signal molecules. **(B)** The expression level of SlSKIP1b with the treatments of defense-related signal molecules.

The function analysis of *SlSKIPs* was completed to reveal their probable biological roles in resisting against disease. First, this study investigated the *SlSKIP1a* and *SlSKIP1b* expression models responding to the inoculation of *Pst* DC3000 and *B. cinerea*, together with the treatment of defense-related signal molecules, including 1-amino cyclopropane-1-carboxylic acid (ACC, the ET precursor), methyl jasmonate (MeJA), and salicylic acid (SA) in tomato plants. After 72 h of *Pst* DC3000 infection, the *SlSKIP1b* level was notably upregulated by about 6.3 times relative to that in control plants with mimic inoculation, whereas the *SlSKIP1a* level showed no difference after *Pst* DC3000 infection (Figure 1A). In the case of inoculation with *B. cinerea*, the condition was very similar to that in inoculation with *Pst* DC3000. After 48 h of *B. cinerea* infection, the *SlSKIP1b* level prominently increased by about 7.1-folds relative to that in control plants with mimic inoculation, but the *SlSKIP1a* level showed no dramatic difference (Figure 1B). Moreover, none of our selected signal molecules associated with defense affected the *SlSKIP1a* level, while all of those signal molecules triggered *SlSKIP1b* expression (Figures 2A,B). In addition, the *SlSKIP1b* level significantly increased at 12 h after ACC and JA treatments, and its expression was the maximal at 24 h after SA treatment (Figure 2B). As suggested by these data, *SlSKIP* expression might be triggered via *B. cinerea* and *Pst* DC3000 as well as the defense-related signal molecules.

### *SlSKIP* Silencing of Tomato

**FIGURE 3:**
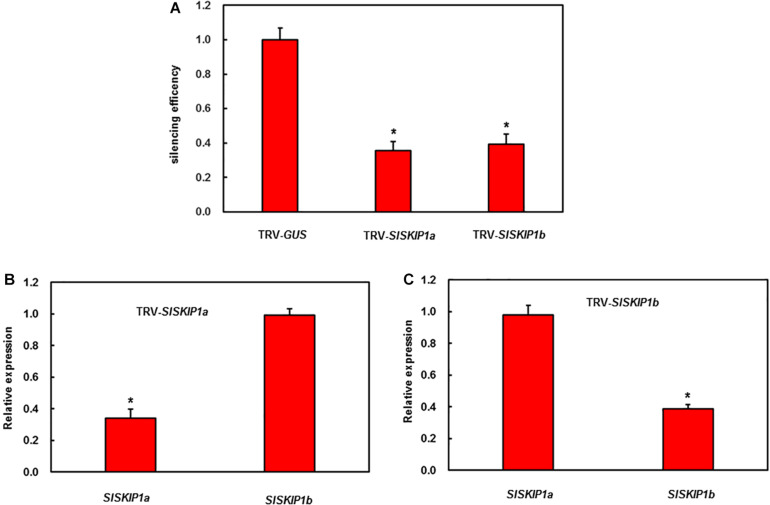
*SlSKIP* gene silencing efficiency along with specificity of plants inoculated with VIGS. **(A)** The *SlSKIP1a* and *SlSKIP1b* silencing efficiency of plants inoculated with TRV-*SlSKIP1a* and those inoculated with TRV-*SlSKIP1b*, respectively. **(B)** *SlSKIP1a* silencing specificity of plants inoculated with TRV-*SlSKIP1a*. **(C)** *SlSKIP1b* silencing specificity of plants inoculated with TRV-*SlSKIP1b*. Data presented are the means ± SD from three independent experiments with biological distinct samples and * above the columns indicate significant differences at *p* < 0.05 level.

For analyzing *SlSKIP* effects on the resistance against disease, the VIGS approach was utilized to manage the endogenous *SlSKIP* expression. Therefore, the *SlSKIP* gene silencing efficiency was checked at first. Then, the normal VIGS protocol was adopted for the 2-week-old tomato seedlings. At 4 weeks later, the silencing efficiency was measured, with plants transfected using the TRV-*PDS* construct being the positive controls. The silencing efficiency of *SlSKIP* genes was evaluated to be 65%, which was used for further functional studies (Figures 3A,B).

### Silencing of *SlSKIP1b* Led to Reduced *B. cinerea* Tolerance

**FIGURE 4:**
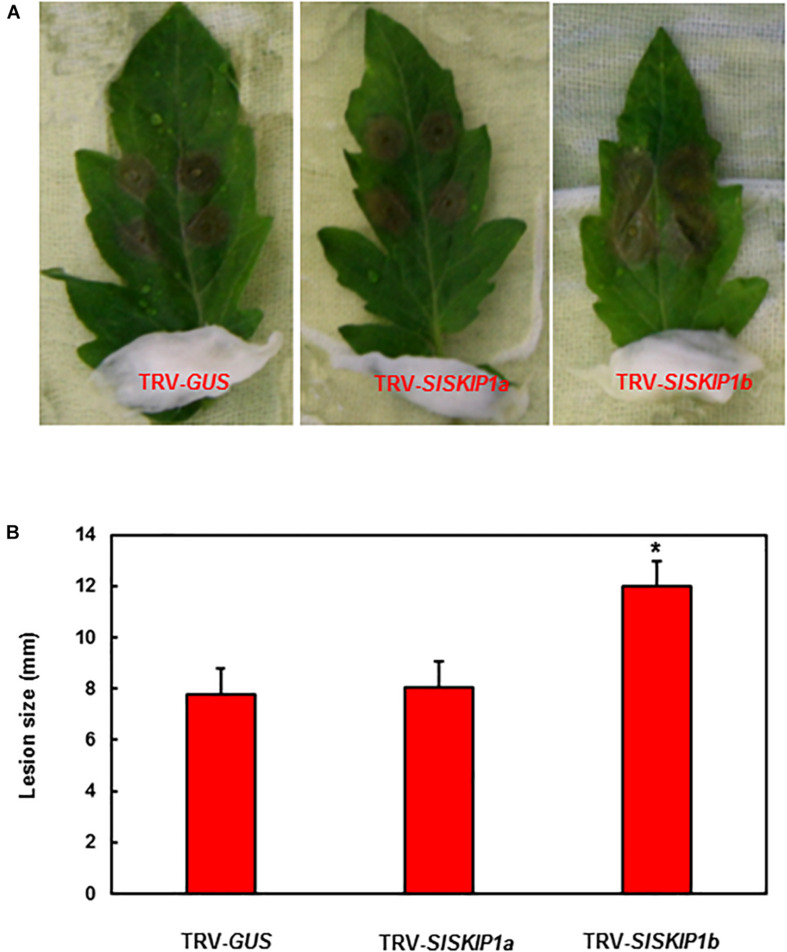
*SlSKIP1b* silencing led to the weakened *B. cinerea* tolerance verified through detached leaf disease assays. Agrobacteria that carried TRV-*SlSKIPs* and TRV-GUS constructs were transfected into the tomato plants of 10 days old, and then leaves were harvested following agroinfiltration for four weeks. **(A)** Disease symptoms of typical leaf samples collected based on plants inoculated with TRV-*SlSKIP* and those inoculated with TRV-GUS. **(B)** Leaf lesion size in plants inoculated with TRV-*SlSKIP* and those inoculated with TRV-GUS. Data presented are the means ± SD from three independent experiments with biological distinct samples and * above the columns indicate significant differences at *p* < 0.05 level.

For studying those potential *SlSKIP* genes’ functions to resist *B. cinerea*, this study applied two distinct approaches, namely, detached leaf and whole plant disease assays for preliminary and further confirmation, respectively. The seedlings of TRV-*SlSKIPs*- and TRV-*GUS*-infiltrated plants were compared for their disease phenotypes and fungal quantity, for the sake of confirming disease phenotype. As obtained from detached leaf disease assays, the leaf lesion size in TRV-*SlSKIP1b*-infiltrated plants prominently elevated by about 54% at 3 days after infection (dpi) (Figure 4A), compared with that in TRV-*GUS*-infiltrated counterparts (Figure 4B). Meanwhile, the leaf lesion size in TRV-*SlSKIP1a*-infiltrated plants (3 dpi) was not significantly different from that of the TRV-*GUS*-infiltrated counterparts (Figures 4A,B).

**FIGURE 5:**
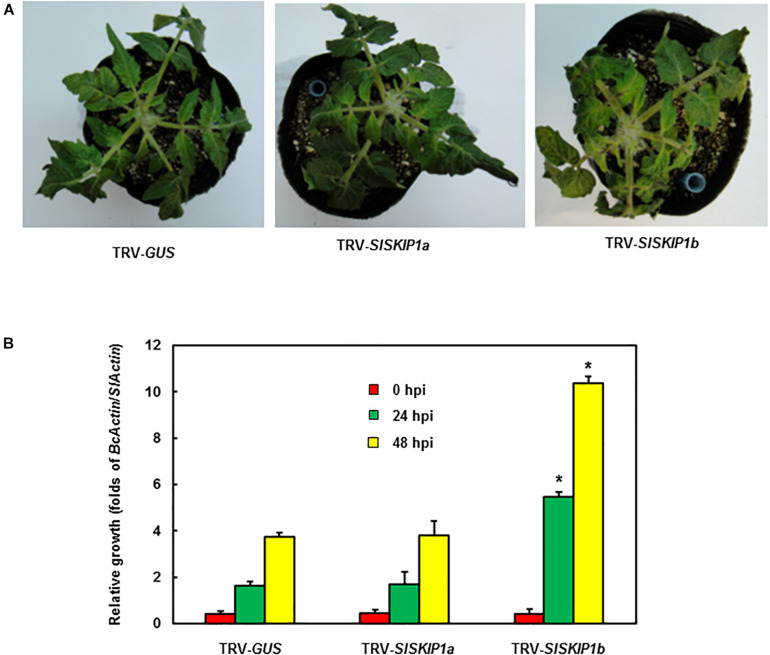
Silencing of *SlSKIP1b* resulted in the weakened *B. cinerea* tolerance evidenced by whole-plant disease assays. **(A)** Disease phenotypes in typical plant leaves inoculated with TRV-*SlSKIP1b* and those inoculated with TRV-*GUS*. After four days of inoculation, the photographs were taken. **(B)** *B. cinerea* growth i*n planta* of plant leaf samples inoculated with TRV-*SlSKIP1b* and those inoculated with TRV-*GUS*. Data presented are the means ± SD from three independent experiments with biological distinct samples and * above the columns indicate significant differences at *p* < 0.05 level.

For further confirming the above finding, whole-plant disease assays were conducted to estimate disease phenotype and test *B. cinerea* fungal growth *in planta* of plants inoculated with TRV-*SlSKIP*. According to Figure 5A, plants infiltrated with TRV-*GUS* showed mild disease symptom compared with those inoculated with TRV-*SlSKIP1b*, while those inoculated with TRV-*SlSKIP1a* displayed no difference from those inoculated with TRV-*GUS* at 5 dpi. At 24 and 48 hpi, the *B. cinerea* growth *in planta*, which was expressed as *B. cinerea BcActinA* gene transcript level, notably elevated by threefold in leaves of plants inoculated with TRV-*SlSKIP1b* compared with those inoculated with TRV-*GUS* (Figure 5B). While *B. cinerea* growth for plants inoculated with TRV-*SlSKIP1a* did not show any significant difference compared with plants inoculated with TRV-*GUS* (Figure 5B). Collectively, the above findings suggested that *SlSKIP1b* silencing reduced *B. cinerea* tolerance in tomato plants, with excessive *B. cinerea* growth of TRV-*SlSKIP1b*-infiltrated plants.

**FIGURE 6:**
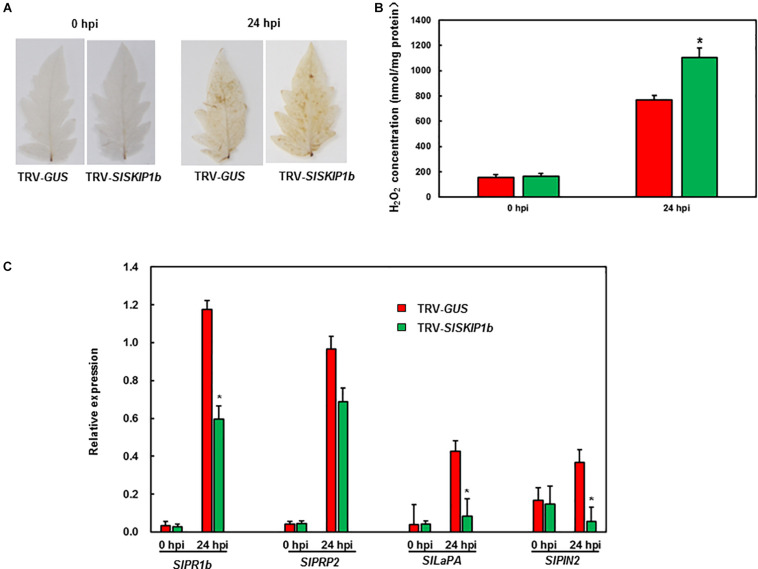
Silencing of *SlSKIP1b* elevated H_2_O_2_ accumulation but downregulated the JA/ET signaling-responsive DRG expression following *B. cinerea* inoculation. At 4 weeks following VIGS inoculation, 2 × 10^5^ spores/mL pore suspension was used to spray on the leaf surface through whole-plant disease assays, then leaves were harvested at 24 h following infection. **(A)** H_2_O_2_ accumulation in plants inoculated with TRV-*SlSKIP1b* and those inoculated with TRV-*GUS* revealed through DAB staining following *B. cinerea* inoculation. **(B)** The H_2_O_2_ concentration in plants inoculated with TRV-*SlSKIP1b* and those inoculated with TRV-*GUS* before and after *B. cinerea* inoculation. The H_2_O_2_ concentration was measured using an H_2_O_2_ kit. **(C)** Specific DRG expression levels in plants inoculated with TRV-*SlSKIP1b* and those inoculated with TRV-*GUS* following *B. cinerea* inoculation. Data presented are the means ± SD from three independent experiments with biological distinct samples and * above the columns indicate significant differences at *p* < 0.05 level.

To gain insights into the probable mechanism by which *SlSKIP1b* silencing led to weakened *B. cinerea* tolerance, this study analyzed the ROS accumulation together with the expression levels of DRGs. Before *B. cinerea* infection, no obvious H_2_O_2_ accumulation was observed in plants inoculated with TRV-*SlSKIP1b* or those inoculated with TRV-*GUS*, but H_2_O_2_ accumulation significantly increased at 24 h following *B. cinerea* inoculation (Figure 6A). The H_2_O_2_ concentration was further measured. The results showed that the H_2_O_2_ concentration in plants inoculated with TRV-*SlSKIP1b* was much higher than that of the ones inoculated with TRV-*GUS* after *B. cinerea* infection, but there was no significant difference before *B. cinerea* infection (Figure 6B). Similarly, *SlPRP2* and *SlPR1b* (the DRGs responding to the SA signaling) and *SlLapA* and *SlPIN2* (DRGs responding to the JA/ET signaling) were almost the same in plants inoculated with TRV-*SlSKIP1b* as those inoculated with TRV-*GUS* prior to *B. cinerea* inoculation (Figure 6C). *B. cinerea* infection was the primary cause inducing the expression of the above four DRGs, relative to those of uninfected controls. However, at 24 hpi, *SlPR1b* and *SlPRP2* expression slightly decreased, whereas *SlPIN2* and *SlLapA* expression notably reduced in plants inoculated with TRV-*SlSKIP1b*, relative to those TRV-*GUS*-infected counterparts (Figure 6C). Collectively, the above results suggested that *SlSKIP1b* silencing resulted in reduced accumulation of ROS, as well as decreased levels of DRGs responding to the JA/ET signaling after *B. cinerea* inoculation.

### *SlSKIP1b* Silencing Led to Weakened *Pst* DC3000 Tolerance

**FIGURE 7:**
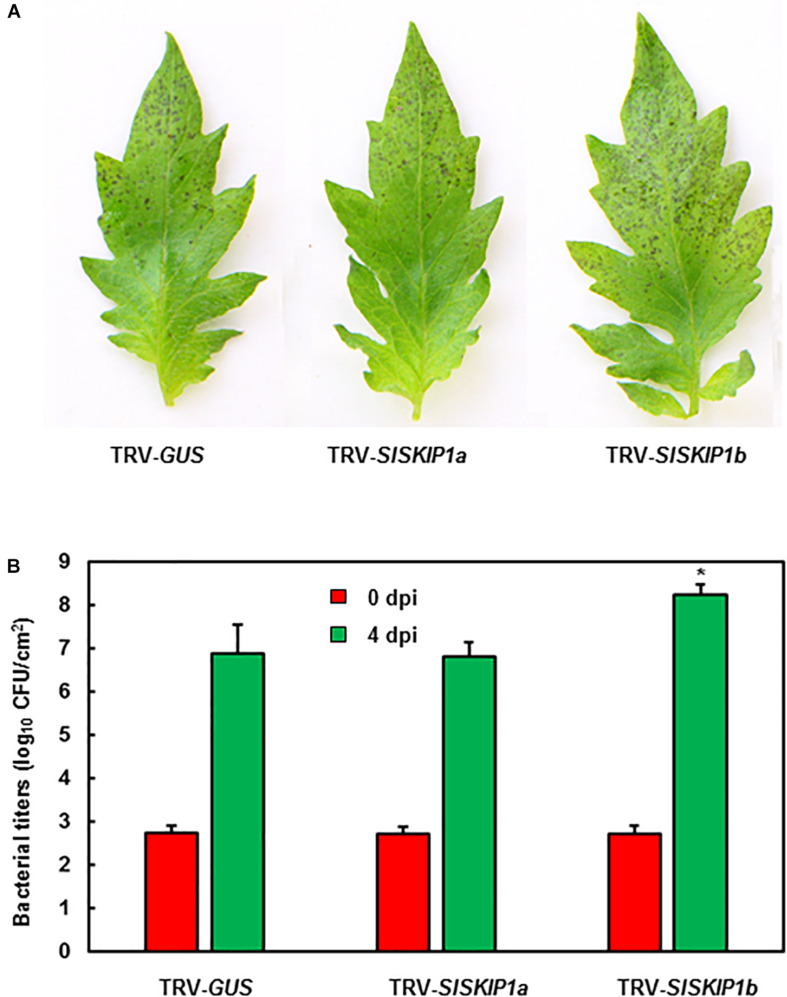
*SlSKIP1b* silencing weakened *Pst* DC3000 tolerance. Agrobacteria that carried TRV-*SlSKIP* and TRV-*GUS* constructs were transfected into the tomato plants 10 days old, at 4 weeks later, the disease assays were performed. **(A)** Disease symptoms in typical plant leaf samples inoculated with TRV-*SlSKIPs* and those inoculated with TRV-*GUS* at 4 days following *Pst* DC3000 infection. **(B)** Bacterial quantity in plant leaf samples inoculated with TRV-*SlSKIPs* and those inoculated with TRV-*GUS*. Leaves were harvested at 0 and 4 days following inoculation to measure the bacterial quantity. Data presented are the means ± SD from three independent experiments with biological distinct samples and * above the columns indicate significant differences at *p* < 0.05 level.

The potential *SlSKIP1b* functions in the resistance against *Pst* DC3000 was further studied. The disease phenotype and *in planta* bacterial quantity of plants inoculated with TRV-*SlSKIPs* were compared with those of plants inoculated with TRV-*GUS*. Differences in disease symptom and bacterial growth at 3 dpi were not significant between plants inoculated with TRV-*SlSKIP1a* and those inoculated with TRV-*GUS* (Figures 7A,B), indicating that *SlSKIP1a* did not possibly participate in the *Pst* DC3000 tolerance. Plants inoculated with TRV-*SlSKIP1b* showed serious disease symptom relative to those inoculated with TRV-*GUS* (Figure 7A), and the bacterial population at 4 dpi was about 20 times higher than that in control (Figure 7B). The above findings demonstrated the effect of *SlSKIP1b* silencing on reducing *Pst* DC3000 tolerance of tomato.

**FIGURE 8:**
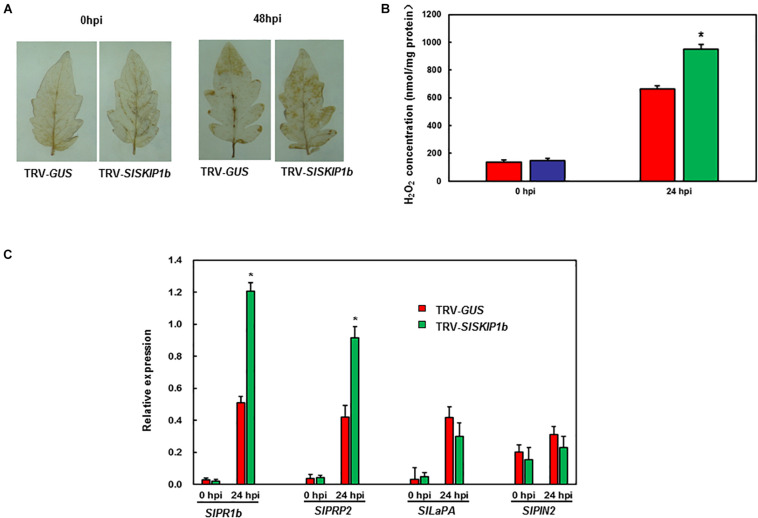
Silencing of *SlSKIP1b* affected H_2_O_2_ accumulation and SA signaling-responsive DRG expression following *Pst* DC3000 inoculation. The *Pst* DC3000 suspension (OD_600_ = 0.0002) was used to infect plants at negative pressure in the whole-plant disease assays 4 weeks following VIGS inoculation. Leaves were harvested to analyze the H_2_O_2_ accumulation together with DRG expression following infection. **(A)** H_2_O_2_ accumulation in plants inoculated with TRV-*SlSKIP1b* and those inoculated with TRV-*GUS* following *Pst* D3000 inoculation measured through DAB staining. **(B)** The H_2_O_2_ concentration in plants inoculated with TRV-*SlSKIP1b* and those inoculated with TRV-*GUS* before and after *Pst* DC3000 inoculation. The H_2_O_2_ concentration was measured using an H_2_O_2_ kit. **(C)** Specific DRG expression in plants inoculated with TRV-*SlSKIP1b* and those inoculated with TRV-*GUS* following *Pst* DC3000 inoculation. Data presented are the means ± SD from three independent experiments with biological distinct samples and * above the columns indicate significant differences at *p* < 0.05 level.

For exploring the potential mechanism of action by which *SlSKIP1b* silencing affected *Pst* DC3000 tolerance, the ROS accumulation together with DRGs expression was examined subsequently. Prior to *Pst* DC3000 infection, there was no distinct H_2_O_2_ accumulation observed in leaves from plants inoculated with TRV-*SlSKIP1b* and TRV-*GUS* (Figure 8A). Compared with controls, plants inoculated with TRV-*SlSKIP1b* showed obvious H_2_O_2_ accumulation at 3 dpi (Figure 8A). We also measured the H_2_O_2_ concentration. As shown in Figure 8B, the H_2_O_2_ concentration in TRV-*SlSKIP1b* seedlings was much higher than that of TRV-*GUS* after pathogen infection. There was no significant difference in *SlPRP2*, *SlPR1b*, *SlPIN2*, or *SlLapA* expression in plants inoculated with TRV-*SlSKIP1b* relative to those inoculated with TRV-*GUS* prior to *Pst* DC3000 inoculation (Figure 8C). Besides, relative to plants inoculated with TRV-*GUS* at 2 dpi, those inoculated with TRV-*SlSKIP1b* showed decreased *SlPR1b* together with *SlPRP2* expression (Figure 8C). However, *SlLapA* or *SlPIN*2 expression of plants inoculated with TRV-*SlSKIP1b* showed no prominent change relative to TRV-*GUS*-infiltrated counterparts at 2 days following *Pst* DC3000 infection (Figure 8C). The above results suggested the effect of *SlSKIP1b* silencing on reducing the SA signaling-responsive DRGs levels by *Pst* DC3000 inoculation.

## Discussion

In this research, two *SlSKIP* genes were identified in tomato, while only one *SKIP* gene was discovered in Arabidopsis. Although it is already known that SKIP is involved in transcription regulation and RNA splicing, thus leading to the regulation of several signaling pathways. There is no direct genetic proof for SKIP functions in the disease resistance of plants.

In our experiment, the *SlSKIP* target gene silencing efficiency was predicted as around 65% (Figure 3A), close to that obtained in our prior works (Li et al., 2014, 2015; Liu et al., 2014; Zhang Y. et al., 2014; Zhang et al., 2016). It is previously reported that pathogen infection may induce *SKIP* expression (Liu, 2015). As found in the present work, both *B. cinerea* and *Pst* DC3000 triggered *SlSKIP1b* expression (Figure 1). Also, *SlSKIP* expression was triggered in response to signal molecules associated with defense (Figure 2), which was consistent with previous study reporting that *OsSKIPa* expression was triggered upon a variety of phytohormone treatments as well as abiotic stress conditions (Hou et al., 2009). The *SlSKIP* genes showed different responses to *B. cinerea* or *Pst* DC3000 inoculation, together with the signal molecules associated with defense, which suggested the potential functions in the *B. cinerea* as well as *Pst* DC3000 tolerance.

The VIGS-based method was adopted to analyze the *SlSKIP* functions in terms of disease resistance. As a result, the silencing of *SlSKIP1b* led to weakened *B. cinerea* (Figures 4, 5) along with *Pst* DC3000 (Figure 7) tolerance. Typically, plants silenced by *SlSKIP1b* displayed the serious disease symptom, together with excessive pathogen growth, which confirmed their reduced *B. cinerea* tolerance (Figures 4, 5). At the same time, the *SlSKIP1b*-silenced plants displayed more severe disease symptoms, along with more bacterial growth, which confirmed the reduced *Pst* DC3000 tolerance (Figure 7). These results were consistent with a previous report that *SKIP* did have a certain function in the resistance to biotic stress. Moreover, *GhSKIP35* has certain functions in the resistance to verticillium wilt in *G. hirsutum* (Liu, 2015).

In this study, the alterations of ROS accumulation and certain specific DRG expression were analyzed to explore the cause of the decreased *B. cinerea* and *Pst* DC3000 tolerance of *SlSKIP1b*-silenced plants. In this experiment, plants silenced by *SlSKIP1b* showed more H_2_O_2_ accumulation following *B. cinerea* and *Pst* DC3000 inoculation (Figures 6, 8). It is known that late-stage ROS accumulation facilitates disease development resulting from the necrotrophic pathogens (like *B. cinerea*) and (hemi) biotrophic pathogens (like *Pst* DC3000) (Govrin and Levine, 2000; Govrin et al., 2006; Temme and Tudzynski, 2009; Ishiga et al., 2012; Mengiste, 2012). Therefore, the increased ROS content resulting from *SlSKIP1b* silencing was possibly related to the weakened tolerance to *B. cinerea* as well as *Pst* DC3000 of plants silenced by *SlSKIP1b*.

Besides, plants silenced by *SlSKIP1b* had decreased levels of *SlRP1b* (SA-related gene), *SlLapA* (JA-related gene), and *SlPIN2* (JA-related gene) following *B. cinerea* inoculation and increased levels of *SlRP1b* and *SlRPP2* (SA-related gene) following *Pst* DC3000 inoculation (Figures 6C, 8C). It is known that SA-regulated defense responses are good for the infection of necrotrophic pathogen *B. cinerea*, while JA-regulated defense responses are involved in restricting the disease. The inverse model is proposed for (hemi) biotrophic pathogen *Pst* DC3000 (Glazebrook, 2005; Pieterse et al., 2009). We speculated that SKIP1b may not mediate in the antagonistic effects between SA- and JA-signaling pathways. Instead, SKIP1b may involve in these two pathways. So the expression levels of SA-dependent genes and lower JA-dependent genes changed in TRV-*SlSKIP1b* inoculated plants.

The reduced resistance of the *SlSKIP1b*-silenced plants might be induced by the increased ROS accumulation together with changed DRGs levels. However, further physiological and biochemical experiments are required to find out the mechanisms responsible for the altered disease resistance observed in the *SlSKIP1b*-silenced plants.

Our results showed that the silencing of *SKIP1b* led to increased susceptibility to both *Botrytis* and *Pst* DC3000. It consists with previous reports that the silencing of genes will reduce or increase resistance to both *Botrytis* and *Pseudomonas* (Li et al., 2015; Wang et al., 2018,2020).

It is suggested that SKIP plays a role in abiotic stress tolerance. SKIP is involved in the ABA signaling and renders the osmotic resistance in the case of salt stress through regulating AS genes of Arabidopsis (Lim et al., 2010; Feng et al., 2015). *OsSKIPa* positively modulates the stress tolerance of rice by regulating different genes associated with stress in rice at the transcription level (Hou et al., 2009). The interaction of OsSKIP with OsCYP18-2 is essential for regulating genes associated with stress at both transcription and post-transcription levels and for enhancing drought resistance (Lee et al., 2015). The *ZmSKIP* overexpression plants with increased ABA contents exhibit significantly enhanced resistance to drought compared with controls, which suggested that *ZmSKIP* was involved in the regulation of drought resistance by regulating specific gene levels (Wei et al., 2015). Nonetheless, no experiment was carried out to examine the abiotic stress tolerance in this study, and no difference was found between plants inoculated with TRV-*SlSKIP1b* and those inoculated with TRV-*GUS* during the vegetative growth process. This might be consistent with previous report that SKIP participated in regulating certain reproductive stage gene expression at the post-transcription level in *Arabidopsis thaliana* (Wang et al., 2012; Cao et al., 2015; Cui et al., 2017). Unfortunately, this study did not conduct an experiment on plants that had entered the reproductive phase. In our future studies, experiments should be performed to examine the resistance to abiotic stress and plants of the reproductive stag.

## Data Availability Statement

The raw data supporting the conclusions of this article will be made available by the authors, without undue reservation.

## Author Contributions

HZ, LY, MJ, and FS carried out most of the experiments. MJ and HZ designed the experiments and wrote the manuscript. All authors read and approved the final manuscript.

## Conflict of Interest

The authors declare that the research was conducted in the absence of any commercial or financial relationships that could be construed as a potential conflict of interest.
