# Supplementary material for: SKIP Silencing Decreased Disease Resistance Against Botrytis cinerea and Pseudomonas syringae pv. tomato DC3000 in Tomato
Source: Front Plant Sci. 2020 Dec 14;11:593267. doi: 10.3389/fpls.2020.593267 (PMC7767821; doi:10.3389/fpls.2020.593267)
Supplement: Supplementary file 1 [file Data_Sheet_1.pdf]

frontiers

Supplementary Material

Table 1 The detail of *SKIPs*

Genes	accessions number	Length of gene(bp)
SKIP1a	XM_004251580.4	1488
SKIP1b	XM_004250540.4	1257

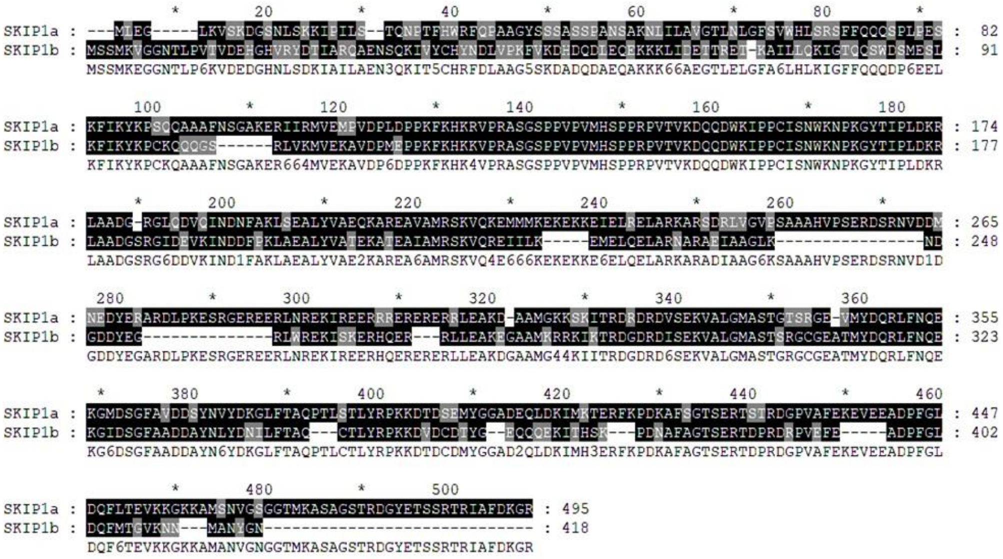

Figure 1 Comparison of the deduced amino acid sequences of *SlSKIPs*. Black colour shows the same amino acid sequences.

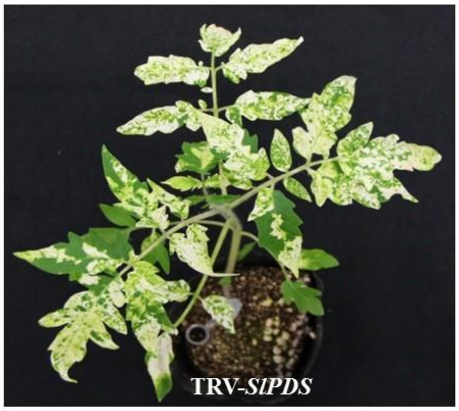

Figure 2 The image of the TRV-*PSD*
